# Mortality risk associated with acetylcholinesterase inhibitor use in Parkinson’s disease dementia according to sex and age at disease onset: a nationwide cohort study

**DOI:** 10.4178/epih.e2026015

**Published:** 2026-04-11

**Authors:** Bora Yoon, Hwa Jung Kim

**Affiliations:** 1 Department of Neurology, Seoul St. Mary’s Hospital, College of Medicine, The Catholic University of Korea, Seoul, Korea; 2 Konyang University Myunggok Medical Research Institute, Daejeon, Korea; 3 Department of Medical Informatics & Statistics, Asan Medical Center, Ulsan University College of Medicine, Seoul, Korea

**Keywords:** Parkinson disease, Dementia, Acetylcholinesterase inhibitor, Mortality, Sex

## Abstract

**OBJECTIVES:**

Dementia increases mortality risk; however, most studies evaluating acetylcholinesterase inhibitors (AChEIs) have focused on Alzheimer’s disease. The survival effects of AChEIs in Parkinson’s disease dementia (PDD) remain unclear. This study evaluated the association between AChEI use and mortality in PDD, stratified by sex and age at Parkinson’s disease (PD) onset.

**METHODS:**

This retrospective cohort study used data from the Korean National Health Insurance Service from January 2002 to December 2021. Patients diagnosed with PDD after PD onset were included. Propensity score matching (2:1) was performed to match AChEI users with non-users. Kaplan–Meier survival analyses and subgroup analyses were conducted according to sex and age at PD onset.

**RESULTS:**

AChEI use was associated with a 24% reduction in mortality risk (hazard ratio [HR], 0.76; 95% confidence interval [CI], 0.74 to 0.78; p<0.001). The survival benefit persisted throughout follow-up and was more pronounced in females (HR, 0.71; 95% CI, 0.69 to 0.74) than in males (HR, 0.83; 95% CI, 0.80 to 0.86). In late-onset PDD, AChEI use was associated with a 26% reduction in mortality (HR, 0.74; 95% CI, 0.72 to 0.76), whereas no significant association was observed in young-onset PDD (HR, 1.02; 95% CI, 0.92 to 1.33; p=0.665). Survival outcomes were comparable between donepezil and rivastigmine users.

**CONCLUSIONS:**

AChEI use improved survival in patients with PDD, particularly in females and in those with late-onset PD, with the greatest benefit observed during early and medium-term follow-up periods. These findings suggest that AChEIs may confer a survival advantage in PDD regardless of the specific agent used.

## GRAPHICAL ABSTRACT

[Fig f4-epih-48-e2026015]

## Key Message

• The use of AChEIs was associated with a lower risk of mortality in patients with PDD in a nationwide cohort.

• The survival advantage was particularly evident among females and those with late-onset PD.

• These findings suggest that tailored use of AChEIs may improve long-term outcomes in PDD.

## INTRODUCTION

Parkinson’s disease (PD) is one of the most common neurodegenerative disorders worldwide, and its burden continues to increase because of population aging, prolonged disease duration, and environmental factors. Over the past several decades, the global prevalence of PD has more than doubled, underscoring its substantial socioeconomic impact [1]. Dementia frequently develops in patients with PD, and the incidence of Parkinson’s disease dementia (PDD) increases markedly over time. Estimates suggest that 3–30% of individuals develop PDD within 5 years of PD diagnosis, and this proportion exceeds 80% after 20 years [2]. A meta-analysis reported an annual incidence rate of PDD of 4.45 per 100 person-years at risk, with patients with PD being 3.25 times more likely to develop dementia than healthy controls [3].

Patients with PD have a substantially higher mortality risk than the general population [4-8]. In a study using data from the Korean National Health Insurance Service (KNHIS), the adjusted hazard ratio (HR) for mortality among patients with PD was 2.96, with a 10-year mortality rate of 47.9%, compared with 20.3% in controls. The leading causes of death in patients with PD include neurological, circulatory, and respiratory diseases [5]. Global mortality rates associated with PD have also increased over time, with higher rates observed in males and older adults [4]. Among patients with PD, the presence of dementia further increases mortality risk [5]. Mortality rates are particularly high in patients with dementia, although they vary across dementia subtypes [9,10]. For example, PDD has an HR of 1.47 for mortality, which is higher than that of mixed Alzheimer’s and vascular dementia (HR, 1.32) but lower than that of frontotemporal dementia (HR, 1.91) [10]. These findings underscore the substantial contribution of dementia to reduced survival in PD.

Acetylcholinesterase inhibitors (AChEIs), which are commonly prescribed for Alzheimer’s disease (AD) to slow cognitive decline, have been associated with reduced all-cause mortality in patients with AD [11,12]. Their potential survival effects have also been examined in other dementia subtypes, including AD, vascular dementia, and dementia with Lewy bodies [12,13]; however, evidence specific to PDD remains limited [13]. Cholinergic deficits play a central role in the cognitive dysfunction observed in PDD [14], and clinical guidelines recommend AChEIs to improve cognitive symptoms [15]. Nevertheless, the potential survival benefit of AChEIs in patients with PDD has not been well characterized.

Using the KNHIS database, this study investigated whether long-term AChEI use was associated with improved survival in patients with PDD. By examining differences according to sex and age at PD onset, we aimed to provide detailed insights into the survival impact of AChEIs in a large, real-world cohort.

## MATERIALS AND METHODS

### Data source

This retrospective cohort study used data from the KNHIS, a nationwide database that includes medical records for approximately 97% of the Korean population. The dataset contains information on demographics, socioeconomic status, diagnoses, treatments, medications, and healthcare costs from January 1, 2002, to December 31, 2021. Diagnoses were recorded using the International Classification of Diseases, 10th revision (ICD-10) [16]. In Korea, PD is classified as a rare and intractable disease (RID) that qualifies for governmental financial support. After confirmation by a qualified clinician, patients are registered with both the ICD-10 code for PD (G20) and the RID code (V124). In this study, PD cases were identified using the KNHIS RID registry.

### Study population

Patients diagnosed with PD (ICD-10 code G20 and RID code V124) between January 2002 and December 2021 were eligible. PDD was defined as dementia diagnosed after the initial PD diagnosis, based on ICD-10 codes F00–F03 and G30.

From an initial cohort of 250,357 patients with PD, the following exclusion criteria were applied: (1) age <40 years or >90 years at the index date (n=4,731); (2) PD diagnosis before January 1, 2005, to allow for a 3-year washout period (n=15); (3) discontinuation of PD claims within 90 days of the index date (n=21,277); (4) no recorded dementia diagnosis during the observation period (n=69,732); (5) pre-existing dementia (n=65,983); (6) only 1 claim for PDD (n=9,647); or (7) AChEI prescriptions before dementia diagnosis (n=3,728). After these exclusions, the final cohort comprised 75,244 patients with PDD, including 57,942 AChEI users and 17,302 non-users.

**Figure 1.**
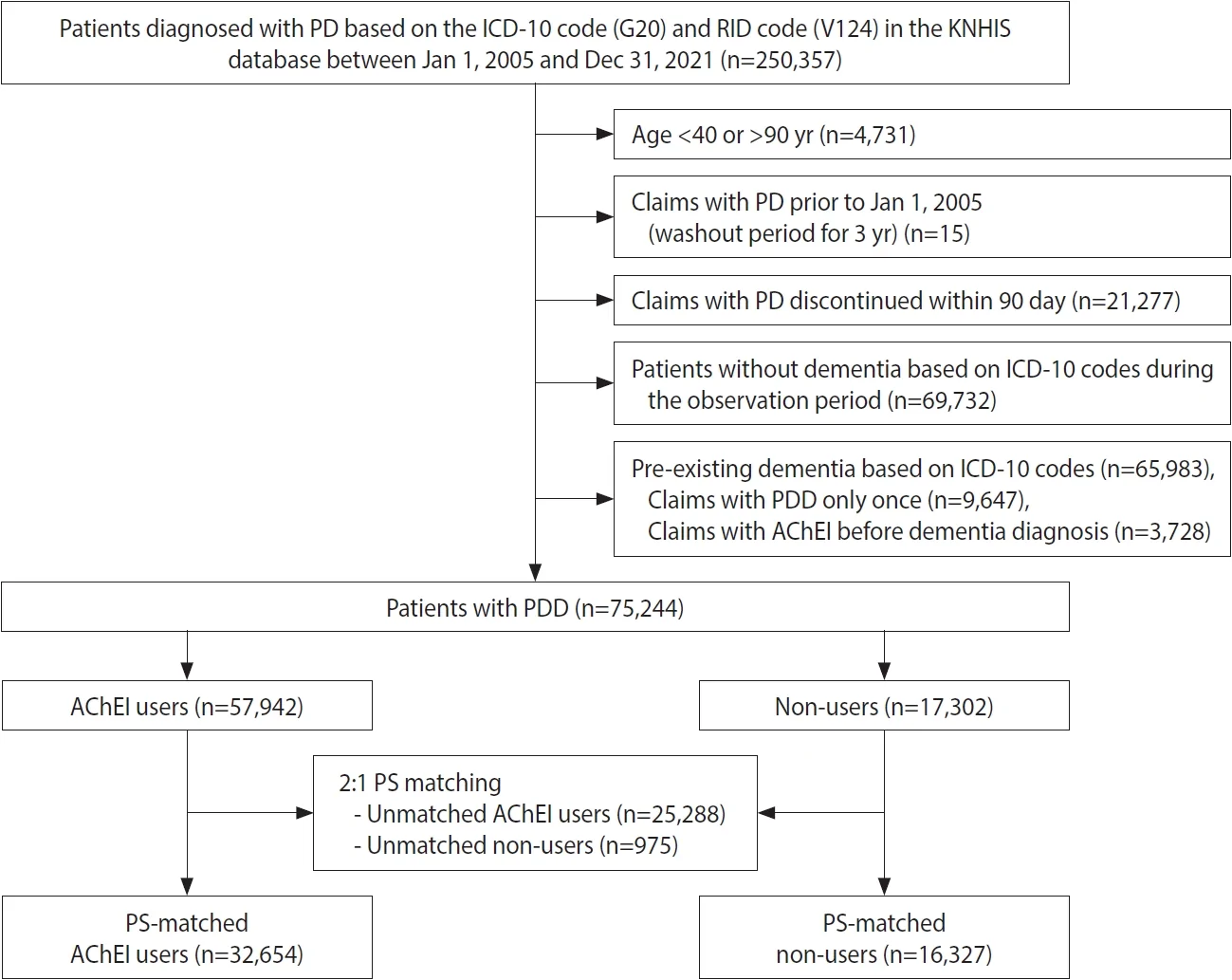
Flowchart of the study population. ICD-10, International Classification of Diseases, 10th revision; RID, rare and intractable disease; KNHIS, Korean National Health Insurance Service; PD, Parkinson’s disease; PDD, Parkinson’s disease dementia; AChEI, acetylcholinesterase inhibitor; PS, propensity score.

To reduce selection bias, propensity score matching (PSM) [17] was performed at a 2:1 ratio, matching AChEI users with non-users using nearest-neighbor matching with a caliper of 0.01. Because mortality was the outcome, the following variables were considered: sex, age, year of diagnosis, region of residence, income quintile, and Charlson comorbidity index (CCI) score [18]. These variables were assessed at 2 time points: PD diagnosis and PDD diagnosis. Accordingly, the propensity score model included: (1) sex; (2) age at PD diagnosis (modeled as a continuous variable and categorized into 5 groups) and age at PDD diagnosis (categorized); (3) year of PD and PDD diagnosis; (4) region of residence at PD and PDD diagnosis; (5) income quintile at PD and PDD diagnosis; and (6) CCI score at PD diagnosis. After matching, the analytic cohort comprised 32,654 AChEI users and 16,327 non-users with PDD (Figure 1).

### Definition of acetylcholinesterase inhibitors and clinical variables

AChEIs were identified using the Anatomical Therapeutic Chemical classification system, including donepezil (N06DA02), rivastigmine (N06DA03), and galantamine (N06DA04). Both inpatient and outpatient prescriptions were included. Patients were classified as AChEI users if they were prescribed any AChEI during the follow-up period. To ensure consistent exposure, only patients who received continuous AChEI prescriptions for at least 3 months were included in the analysis. Pre-existing comorbidities within 1 year were assessed using the CCI, along with additional risk factors such as hypertension (I10–I13, I15), dyslipidemia (E78), and femur fractures (S72).

### Statistical analysis

Covariate balance before and after PSM was assessed using standardized mean differences (SMDs), with values >0.1 indicating meaningful imbalance [19]. Categorical variables are presented as counts and percentages, and continuous variables as means with standard deviations (SDs). Group comparisons used the chi-square test for categorical variables and Student’s t-test for continuous variables.

Cox proportional hazards regression models were used to estimate hazard ratios (HRs) for mortality, comparing AChEI users with non-users. Kaplan–Meier survival curves were generated to visualize mortality differences. Subgroup analyses were stratified by sex and age at PD onset: young-onset (YO; <60 years) and late-onset (LO; ≥60 years).

All analyses were performed using SAS version 9.4 (SAS Institute Inc., Cary, NC, USA) and R version 4.0.5 (R Foundation for Statistical Computing, Vienna, Austria). Statistical significance was set at p-value <0.05.

### Ethics statement

All procedures involving human participants adhered to the ethical standards of the Committee on Human Experimentation of our institution and to the Declaration of Helsinki (1975). The study was approved by the Institutional Review Board of Konyang University Hospital (IRB No. KYUH 2023-02-018). The requirement for informed consent was waived because the data were de-identified and provided by the KNHIS under strict confidentiality regulations.

## RESULTS

### Baseline characteristics of the study population

Table 1 summarizes baseline clinical characteristics of the study population according to AChEI use in the PDD cohort. After PSM, the median follow-up duration for the matched cohort (n=48,981) was 4.01 years (interquartile range [IQR], 2.13–6.81). AChEI users (n=32,654) had a median follow-up of 4.38 years (IQR, 2.46–7.30), whereas non-users (n=16,327) had a shorter median follow-up of 3.24 years (IQR, 1.49–5.82). The mean age was 71.7 years at PD diagnosis and 75.0 years at PDD diagnosis in both groups (Table 1). After matching, no meaningful differences were observed between groups in sex, region of residence, income quintile, CCI score, or comorbidities, except for renal disease (2.4% in the AChEI group vs. 2.8% in the non-user group, p=0.004). However, the SMD for renal disease was 0.027, below the 0.1 threshold, indicating that the imbalance was not meaningful (Table 1).

### Prescription of acetylcholinesterase inhibitors

Among AChEI users, the median treatment duration was 21.1 months (IQR, 6.4–46.5) in the overall cohort and 19.4 months (IQR, 6.0–43.7) after PSM. After matching, 65.4% of patients (n=21,358) initiated donepezil, 29.5% (n=9,633) initiated rivastigmine, and 4.5% (n=1,477) initiated galantamine; this distribution was similar to that observed before PSM (donepezil, 66.5% [n=38,560]; rivastigmine, 28.6% [n=16,571]; galantamine, 4.4% [n=2,521]). In addition, 186 patients (0.6%) after PSM and 290 patients (0.5%) before PSM were prescribed more than 1 AChEI.

### Effect of acetylcholinesterase inhibitor use on mortality

**Figure 2.**
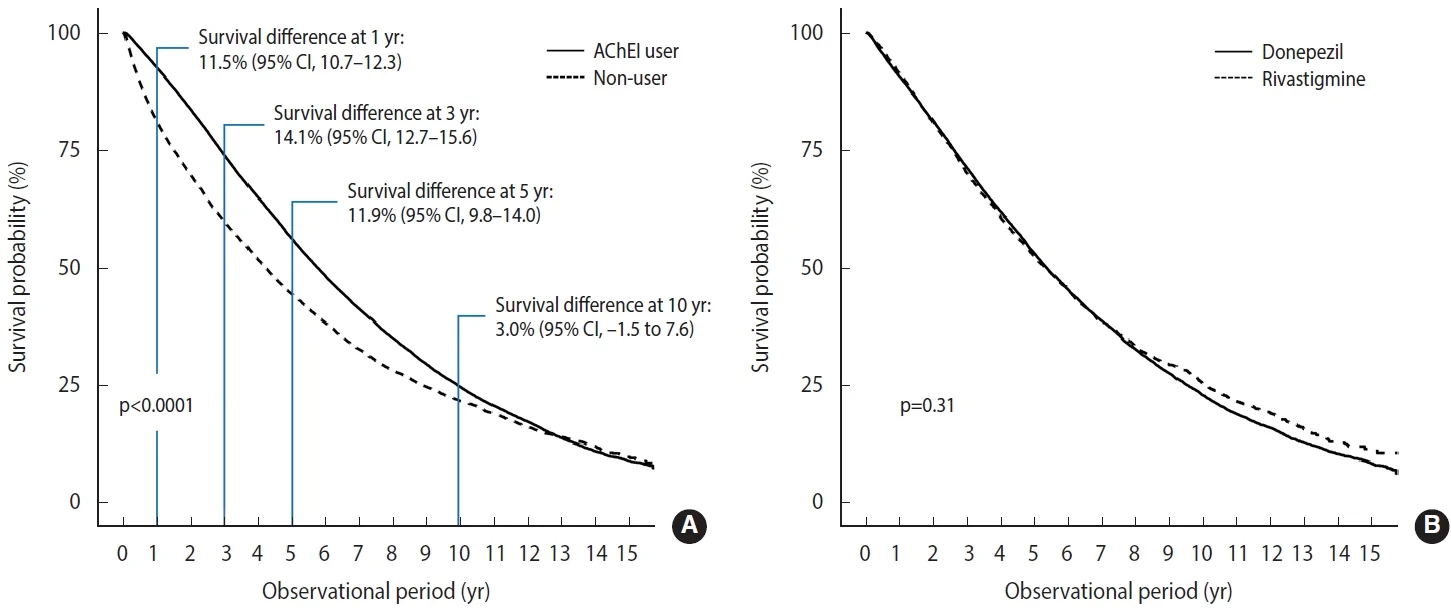
Kaplan–Meier survival curves for overall mortality among patients with PDD. (A) Comparison between AChEI users and non-users. A consistent survival advantage was observed among AChEI users throughout follow-up. Although the magnitude of the survival difference decreased over time, AChEI users maintained a statistically significant survival advantage, particularly during the early and middle follow-up periods. (B) Comparison between donepezil and rivastigmine users. No significant difference in mortality was observed between the two AChEI subgroups. AChEI, acetylcholinesterase inhibitor; PDD, Parkinson’s disease dementia; CI, confidence interval.

AChEI use was associated with a significant reduction in mortality among patients with PDD (Table 2). Overall, AChEI use was associated with a 24% reduction in the risk of death (HR, 0.76; 95% confidence interval [CI], 0.74 to 0.78; p<0.001) (Table 2). Kaplan–Meier survival curves comparing AChEI users and non-users (Figure 2A) demonstrated a consistent survival advantage for AChEI users throughout follow-up. The absolute differences in survival between AChEI users and non-users at key time points were 11.5% (95% CI, 10.7 to 12.3) at 1 year, 14.1% (95% CI, 12.7 to 15.6) at 3 years, 11.9% (95% CI, 9.8 to 14.0) at 5 years, and 3.0% (95% CI, −1.5 to 7.6) at 10 years. Although the survival advantage attenuated over time, the benefit remained statistically significant during the early and medium-term follow-up periods.

A comparison of donepezil and rivastigmine users (Figure 2B) revealed no significant difference in overall mortality between the 2 groups, suggesting that the association between AChEI use and survival was consistent across agents.

**Figure 3.**
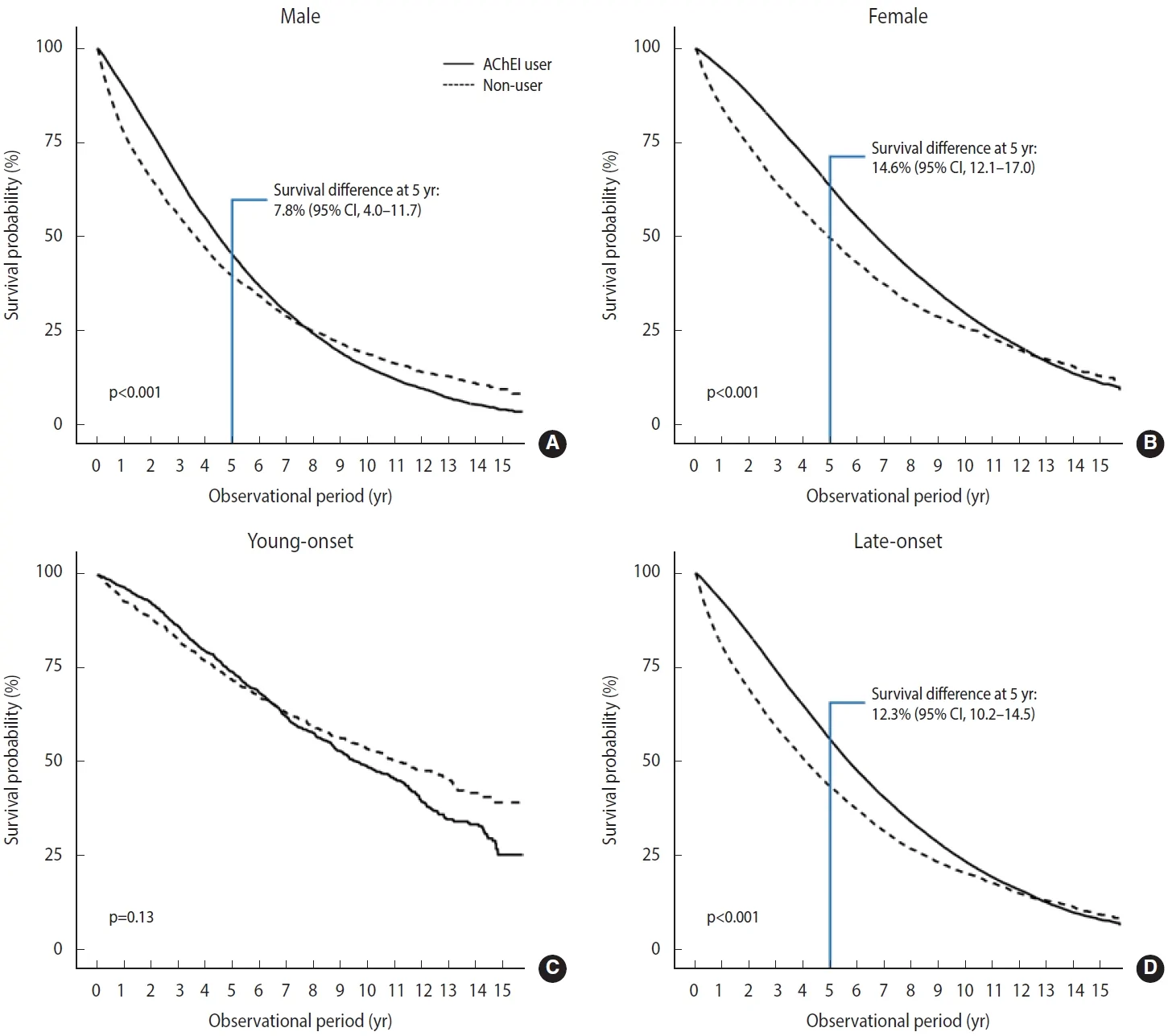
Subgroup analyses of mortality in patients with PDD by sex and age at PD onset. Differences by sex: Kaplan–Meier survival curves for males (A) and females (B). AChEI users exhibited lower mortality than non-users in both sexes, with a more pronounced and sustained survival advantage observed in females. Differences by age at PD onset: Kaplan–Meier survival curves for young-onset (C) and late-onset (D) PDD. While no significant survival difference was observed in the young-onset group, AChEI use was associated with a clear survival advantage in patients with late-onset PDD. AChEI, acetylcholinesterase inhibitor; PDD, Parkinson’s disease dementia; CI, confidence interval.

Sex-stratified analyses showed that both male and female AChEI users had significantly lower mortality than their respective non-user counterparts (Figure 3A and B). However, the magnitude of the association was greater in females (HR, 0.71; 95% CI, 0.69 to 0.74; p<0.001) than in males (HR, 0.83; 95% CI, 0.80 to 0.86; p<0.001) (Table 2). At 5 years of follow-up, the absolute survival difference was 7.8% (95% CI, 4.0 to 11.7) among males (Figure 3A) and 14.6% (95% CI, 12.1 to 17.0) among females (Figure 3B), indicating a more pronounced and sustained survival advantage in females.

The association between AChEI use and survival differed according to age at PD onset. Among patients with YO-PDD, Kaplan–Meier curves (Figure 3C) showed no significant difference in survival between AChEI users and non-users. In contrast, patients with LO-PDD (Figure 3D) demonstrated a substantial survival advantage associated with AChEI use, with an absolute 5-year survival difference of 12.3% (95% CI, 10.2 to 14.5).

Consistent with these findings, AChEI use in LO-PDD was associated with a 26% reduction in mortality (HR, 0.74; 95% CI, 0.72 to 0.76; p<0.001) (Table 2). In contrast, no statistically significant association was observed in YO-PDD (HR, 1.02; 95% CI, 0.92 to 1.33; p=0.665) (Table 2). In multivariable Cox regression analyses, mortality risk increased progressively with advancing age at PD onset, whereas the association between AChEI use and reduced mortality remained consistent across age strata (Supplementary Material 1).

## DISCUSSION

In this nationwide cohort study, AChEI use was associated with a 24% reduction in overall mortality among patients with PDD (HR, 0.76; 95% CI, 0.74 to 0.78). This estimate is comparable to the reduction in all-cause mortality reported in a previous meta-analysis of patients with dementia (adjusted HR, 0.77; 95% CI, 0.70 to 0.84), although that analysis was not specific to PDD [13]. Our findings are also consistent with a systematic review and meta-analysis of 4 trials involving 941 patients with PD, which reported that AChEIs were associated with reduced mortality, slower cognitive decline, and improved behavioral symptoms, without increasing fall risk, worsening motor symptoms, or exacerbating disability [20]. Taken together, these findings support the possibility that AChEIs may influence survival in PDD, potentially through both cognitive stabilization and broader systemic effects.

Several mechanisms may explain the observed association between AChEI use and improved survival in PDD. Given the central role of cholinergic deficits in PDD [14,15], AChEIs enhance cholinergic neurotransmission and thereby support cognitive function. Beyond cognitive effects, AChEIs may influence survival through autonomic and cardiovascular pathways. By increasing vagal tone, lowering heart rate, and potentially reducing arrhythmic events, AChEIs have been hypothesized to decrease the risk of cardiovascular complications, which are major contributors to mortality in dementia [20]. In addition, activation of the “cholinergic anti-inflammatory pathway” may attenuate systemic inflammation and promote cardiovascular stability [21,22]. Epidemiologic studies have reported that AChEI use is associated with a lower risk of myocardial infarction and all-cause mortality in large nationwide dementia cohorts [23]. A recent meta-analysis similarly demonstrated a 37% relative reduction in major adverse cardiovascular events among patients treated with AChEIs [24]. Although these findings do not establish causality, they suggest that modulation of autonomic and inflammatory pathways may partially contribute to the survival advantage observed in our PDD cohort. The importance of comorbidity management is further supported by KNHIS data indicating a 2.5-fold higher mortality risk in patients with PD compared with controls, with comorbidities such as stroke and chronic obstructive pulmonary disease substantially increasing mortality risk [25]. Emerging evidence also suggests that AChEIs may exert neuroprotective effects beyond cholinergic enhancement, potentially slowing dementia progression and reducing complications such as pneumonia, a leading cause of death in this population [13].

Although AChEIs provide therapeutic benefits, they are associated with adverse effects [26-28]. A systematic review and meta-analysis of 48 trials involving 22,845 patients identified anorexia as the most common psychiatric adverse event, followed by agitation, insomnia, and depression [29]. Patients with AD or PDD treated with AChEIs had a higher risk of appetite disturbances, insomnia, and depression than those receiving placebo [29]. Concerns have also been raised regarding a potential increase in fall-related adverse events, which could offset survival advantages in certain patients [30]. These risks may partly explain why some individuals in real-world practice do not initiate or discontinue AChEI therapy, underscoring the importance of individualized treatment decisions that balance potential benefits and harms.

Our subgroup analyses demonstrated that the association between AChEI use and mortality differed according to age at PD onset. Patients with LO-PDD derived a substantial survival benefit from AChEI therapy (HR, 0.74; 95% CI, 0.72 to 0.76), a population that often exhibits a higher prevalence of mixed neuropathologies, including AD and dementia with Lewy bodies [31-33]. Mixed pathologies are common in older adults [34] and are associated with more pronounced cholinergic deficits, which may enhance responsiveness to AChEIs and contribute to improved survival in this group. Furthermore, the higher prevalence of AD among females may enhance the efficacy of AChEIs in female patients with PDD. LO-PDD is also characterized by more rapid progression of cognitive and motor symptoms [35-37], potentially rendering patients more responsive to AChEI treatment during critical phases of disease progression. In contrast, no significant association between AChEI use and mortality was observed in YO-PDD (HR, 1.02; 95% CI, 0.92 to 1.33). This finding should be interpreted cautiously. Mortality risk increases steeply with advancing age at PD onset, resulting in substantially lower baseline mortality among patients with YO-PDD. Patients with YO-PDD typically exhibit more pronounced PD-related pathology and slower cognitive decline [31-33], which may attenuate short- or long-term survival effects of AChEI therapy. It is also possible that greater underlying neurodegeneration at the time of dementia onset in YO-PDD limits responsiveness to cholinergic therapy [38].

Female patients with PDD experienced a more pronounced association between AChEI use and reduced mortality (HR, 0.71; 95% CI, 0.69 to 0.74) than male patients (HR, 0.83; 95% CI, 0.80 to 0.86). Sex-related differences in PD pathophysiology and treatment outcomes are well documented [39]. Females tend to experience faster disease progression, higher mortality, and more non-motor symptoms, such as anxiety and depression, whereas males are more likely to develop greater cognitive decline [39]. These differences may partly account for the observed heterogeneity in survival associations. Another possible explanation involves the neuroprotective effects of estrogen, which may preserve cholinergic function and enhance responsiveness to AChEI therapy, even after menopause [40]. In addition, sex-related psychosocial factors, including differences in health-seeking behavior and treatment adherence, may contribute. Although females are generally more proactive in seeking medical care [41], some studies suggest lower adherence to chronic medications and lower likelihood of receiving guideline-recommended therapies compared with males [42,43]. Because treatment adherence was not directly assessed in this study, these factors remain speculative but may partially explain the observed sex differences.

We observed no significant difference in overall mortality between patients treated with different AChEIs, including donepezil and rivastigmine, suggesting that the association between AChEI therapy and survival was consistent across agents. This finding provides clinicians with flexibility in selecting an AChEI based on individual patient characteristics and tolerability, without concern for differential survival outcomes.

This study has several limitations. First, although indication bias is inherent in observational research, we employed a new-user design and rigorous PSM to enhance comparability between groups. In the absence of an active pharmacologic alternative for PDD, a non-user control group was used, and matching incorporated extensive covariates assessed at both PD and PDD diagnosis to account for disease progression. Additional multivariable regression and subgroup analyses were performed to further reduce residual confounding. The consistency of findings across most strata supports the robustness of the observed associations, although residual confounding cannot be excluded. Second, reliance on administrative claims data may have resulted in diagnostic misclassification, particularly in patients with overlapping PD and dementia features, despite strict inclusion and exclusion criteria. Third, the dataset lacked detailed clinical information, including disease severity, cognitive trajectories, and genetic factors. Although treatment duration was reported, claims data do not permit assessment of medication adherence, cumulative exposure, or dose–response relationships. Fourth, clinical events that may influence survival, such as falls or fall-related injuries—recognized adverse effects of AChEIs—were not directly evaluated as mediators. Although femur fractures were examined as a proxy for severe falls (Table 1), their low prevalence (1.0% in both groups) precluded their inclusion as major covariates in the final mortality models. Consequently, incident falls occurring after AChEI initiation could not be fully accounted for. Finally, because this study was conducted in a Korean population, generalizability to other racial or ethnic groups may be limited. Despite these limitations, this study has important strengths. It represents one of the largest population-based cohort studies examining AChEI use in PDD, leveraging comprehensive nationwide data from the KNHIS. Stratification by sex, age at PD onset, and AChEI type provides clinically relevant insights into potential heterogeneity in survival associations.

In conclusion, AChEI use was associated with a significant reduction in mortality among patients with PDD, particularly among females and those with late-onset PD. These findings suggest that continued AChEI therapy may be associated with improved survival in selected subgroups. Further research is needed to clarify the biological mechanisms underlying the differential associations observed across sex and age at disease onset.

## Figures and Tables

**Figure f4-epih-48-e2026015:**
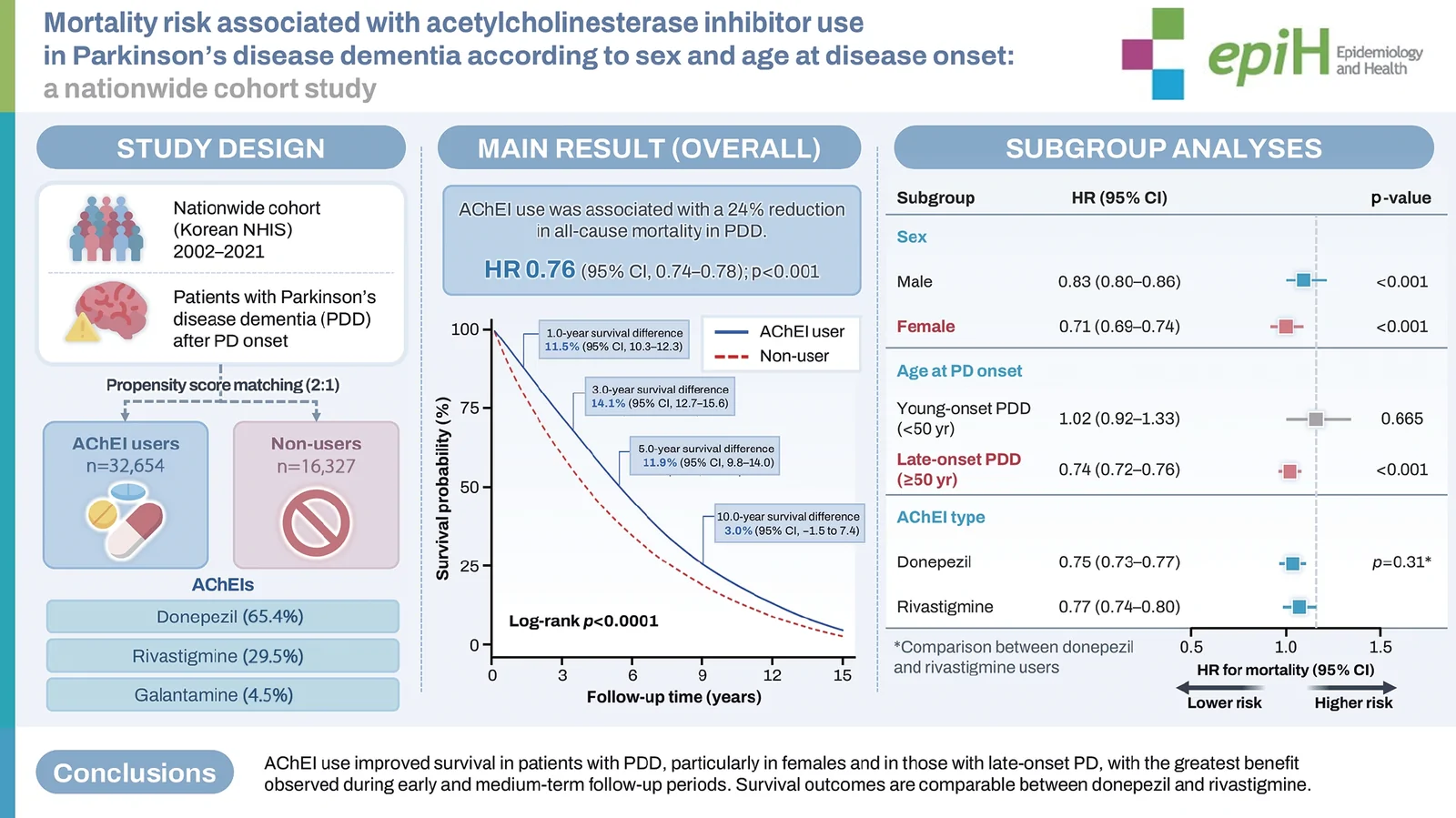


**Table 1.** Characteristics of patients before and after PSM

Characteristics	Before PSM (n=75,244)				After PSM (n=48,981)			
	AChEI user	Non-user	p-value^1^	SMD	AChEI user	Non-user	p-value^1^	SMD
Patients (n)	57,942	17,302			32,654	16,327		
Sex (male)	22,762 (39.3)	7,294 (42.2)	<0.001	0.058	13,426 (41.1)	6,724 (41.2)	0.894	0.001
Mean age at PD diagnosis	72.1±7.5	70.8±9.0	<0.001	0.163	71.7±7.9	71.7±8.1	0.380	0.008
Age group at PD diagnosis			<0.001	0.224			0.813	0.012
40–49	397 (0.7)	367 (2.1)			167 (0.5)	92 (0.6)		
50–59	3,178 (5.5)	1,684 (9.7)			2,315 (7.1)	1,197 (7.3)		
60–69	15,335 (26.5)	4,787 (27.7)			9,357 (28.7)	4,652 (28.5)		
70–79	30,044 (51.9)	7,731 (44.7)			15,435 (47.3)	7,701 (47.2)		
≥80	8,988 (15.5)	2,733 (15.8)			5,380 (16.5)	2,685 (16.4)		
Mean age at PDD diagnosis	75.4±7.3	74.0±8.9	<0.001	0.168	75.0±7.6	75.0±7.9	0.375	0.008
Age group at PDD diagnosis			<0.001	0.246			0.084	0.027
40–49	119 (0.2)	159 (0.9)			14 (0.0)	12 (0.1)		
50–59	1,463 (2.5)	1,053 (6.1)			868 (2.7)	498 (3.1)		
60–69	9,408 (16.2)	3,524 (20.4)			6,732 (20.6)	3,335 (20.4)		
70–79	29,899 (51.6)	7,633 (44.1)			15,236 (46.7)	7,604 (46.6)		
≥80	17,053 (29.4)	4,933 (28.5)			9,804 (30.0)	4,878 (29.9)		
Residential area			<0.001	0.132			>0.999	0.018
Seoul and capital area^2^	23,005 (39.7)	7,293 (42.2)			13,776 (42.2)	6,840 (41.9)		
Metropolitan city^3^	10,785 (18.6)	3,489 (20.2)			6,453 (19.8)	3,245 (19.8)		
Rural area	24,141 (41.7)	6,517 (37.7)			12,425 (38.1)	6,242 (38.2)		
Income quintiles			<0.001	0.080			0.888	0.015
Unclassified	1,137 (2.0)	291 (1.7)			561 (1.7)	281 (1.7)		
Medical Aid	5,608 (9.7)	2,026 (11.7)			3,488 (10.7)	1,799 (11.0)		
Q1 (lowest)	6,527 (11.3)	1,971 (11.4)			3,725 (11.4)	1,847 (11.3)		
Q2	5,064 (8.7)	1,577 (9.1)			2,858 (8.8)	1,461 (8.9)		
Q3	7,048 (12.2)	2,195 (12.7)			4,085 (12.5)	2,054 (12.6)		
Q4	11,237 (19.4)	3,294 (19.0)			6,354 (19.5)	3,135 (19.2)		
Q5 (highest)	21,321 (36.8)	5,948 (34.4)			11,583 (35.5)	5,750 (35.2)		
CCI score	2.3 (2.0)	2.1 (2.0)	<0.001	0.070	2.2 (2.0)	2.2 (2.0)	0.836	0.002
Comorbidities								
Hypertension	35,189 (60.7)	9,897 (57.2)	<0.001	0.072	19,291 (59.1)	9,577 (58.7)	0.379	0.009
Dyslipidemia	25,001 (43.1)	6,894 (39.8)	<0.001	0.067	13,523 (41.4)	6,614 (40.5)	0.057	0.018
MI	916 (1.6)	273 (1.6)	>0.999	<0.001	512 (1.6)	268 (1.6)	0.566	0.006
CHF	4,901 (8.5)	1,277 (7.4)	<0.001	0.040	2,609 (8.0)	1,254 (7.7)	0.238	0.012
PVD	12,717 (21.9)	3,432 (19.8)	<0.001	0.052	6,761 (20.7)	3,338 (20.4)	0.510	0.006
CVD	22,330 (38.5)	6,358 (36.7)	<0.001	0.037	12,228 (37.4)	6,081 (37.2)	0.670	0.004
COPD	15,189 (26.2)	4,172 (24.1)	<0.001	0.048	7,990 (24.5)	4,033 (24.7)	0.580	0.005
DM	17,603 (30.4)	4,849 (28.0)	<0.001	0.052	9,474 (29.0)	4,658 (28.5)	0.270	0.011
Renal disease	1,438 (2.5)	471 (2.7)	0.082	0.015	781 (2.4)	462 (2.8)	0.004	0.027
Cancer	3,021 (5.2)	915 (5.3)	0.714	0.003	1,652 (5.1)	873 (5.3)	0.181	0.013
Femur fracture	567 (1.0)	180 (1.0)	0.499	0.006	311 (1.0)	169 (1.0)	0.408	0.008

Values are presented as mean±standard deviation or number (%). PSM, propensity score matching; AChEI, acetylcholinesterase inhibitor; SMD, standardized mean difference; PD, Parkinson’s disease; PDD, Parkinson’s disease dementia; CCI, Charlson comorbidity index; MI, myocardial infarction; CHF, congestive heart failure; PVD, peripheral vascular disease; CVD, cerebrovascular disease; COPD, chronic obstructive pulmonary disease; DM, diabetes mellitus.

1 By Student’s *t*-test for continuous variables or chi-square test for categorical variables.

2 Seoul, Incheon, and Gyeonggi-do.

3 Busan, Daegu, Daejeon, Gwangju, Sejong, and Ulsan.

**Table 2.** Association between AChEI use and mortality in the matched PDD cohort: total and subgroup analyses by sex and age at PD onset

Subgroups	HR (95% CI)	p-value
Overall	0.76 (0.74, 0.78)	<0.001
Sex		
Male	0.83 (0.80, 0.86)	<0.001
Female	0.71 (0.69, 0.74)	<0.001
Age at PD onset		
Young-onset PDD	1.02 (0.92, 1.33)	0.665
Late-onset PDD	0.74 (0.72, 0.76)	<0.001

AChEI, acetylcholinesterase inhibitor; PDD, Parkinson’s disease dementia; PD, Parkinson’s disease; HR, hazard ratio; CI, confidence interval.

## Data Availability

Eligible researchers can access confidential data through the Korea National Health Insurance (NHI) Sharing Service Institutional Data Access/Ethics Committee (https://nhiss.nhis.or.kr/bd/ay/bdaya001iv.do). To access the NHI data-sharing service, researchers must first obtain IRB approval from their institution. Once approved, researchers can request access to the data, and their application will be evaluated by the Korea NHI Sharing Service Institutional Data Access/Ethics Committee. Researchers are responsible for covering the expenses associated with data access and usage. If other researchers wish to obtain data access, they must follow the same application process. The authors had no special access privileges.

## Supplementary materials

Supplementary Material 1. Crude and multivariable-adjusted hazard ratios for mortality according to AChEI use and baseline characteristics in the matched PDD cohort
