# Supplementary material for: Mortality risk associated with acetylcholinesterase inhibitor use in Parkinson’s disease dementia according to sex and age at disease onset: a nationwide cohort study
Source: Epidemiol Health. 2026 Apr 11;48:e2026015. doi: 10.4178/epih.e2026015 (PMC13503063; doi:10.4178/epih.e2026015)
Supplement: Supplementary file 1 [file epih-48-e2026015-Supplementary-1.docx]

**Supplementary Material 1.** Crude and multivariable-adjusted hazard ratios for mortality according to AChEI use and baseline characteristics in the matched PDD cohort

|  | crude HR (95% CI) | p | adjusted HR (95% CI) | p |
| --- | --- | --- | --- | --- |
| Model 1: Multivariable Analysis with Decades of Age at Onset | | | | |
| AChEI use | 0.76 (0.74-0.78) | <0.001 | 0.74 (0.73-0.76) | <0.001 |
| Male Sex (reference: Female) | 1.52 (1.49-1.56) | <0.001 | 1.63 (1.59-1.66) | <0.001 |
| Age at PD onset | 0.71 (0.69-0.74) | <0.001 |  | <0.001 |
| 40-49 years | (reference) |  | (reference) |  |
| 50-59 years | 1.23 (1.00-1.52) | 0.0524 | 1.27 (1.03-1.57) | 0.0242 |
| 60-69 years | 1.61 (1.31-1.97) | <0.001 | 1.74 (1.42-2.13) | <0.001 |
| 70-79 years | 2.40 (1.96-2.94) | <0.001 | 2.71 (2.21-3.32) | <0.001 |
| ≥ 80 years | 3.49 (2.84-4.28) | <0.001 | 4.01 (3.27-4.92) | <0.001 |
| Model 2: Multivariable Analysis with Binary Age Classification (<60 vs. ≥ 60) | | | | |
| AChEI use | 0.76 (0.74-0.78) | <0.001 | 0.76 (0.74-0.78) | <0.001 |
| Male | 1.52 (1.49-1.56) | <0.001 | 1.55 (1.52-1.59) | <0.001 |
| Age of PD onset |  |  |  |  |
| < 60 years | 0.54 (0.51-0.57) | <0.001 | 0.50 (0.48-0.53) | <0.001 |
| ≥ 60 years | (reference) |  | (reference) |  |

HR, hazard ratio; CI, confidence interval; AChEI, Acetylcholinesterase inhibitor; PD, Parkinson’s disease.
